# Association between social jetlag food consumption and meal times in patients with obesity-related chronic diseases

**DOI:** 10.1371/journal.pone.0212126

**Published:** 2019-02-12

**Authors:** Maria Carliana Mota, Catarina Mendes Silva, Laura Cristina Tibiletti Balieiro, Bruna Fernandes Gonçalves, Walid Makin Fahmy, Cibele Aparecida Crispim

**Affiliations:** 1 Faculty of Medicine of the Federal University of Uberlândia, Minas Gerais, Brazil; 2 Hospital e Maternidade Municipal de Uberlândia, Department of Obstetrics, Uberlândia, Brazil; University of Rhode Island, UNITED STATES

## Abstract

Chronic disruption of the synchronous relationship between endogenous and exogenous circadian timing is associated with the development of obesity and metabolic disease. Social jetlag is a measure of circadian misalignment and has been identified as a risk factor for overweight and related diseases. However, the mechanisms involved in this relationship remain underexplored. The objective of this study was to investigate the association between social jetlag and food consumption at late meal timing in patients with obesity-related chronic diseases. This study included 792 individuals (73% female; age 55.9 ± 12.4 years) in which the prevalence of social jetlag (>1h) was 24.4% (n = 194). Participants with social jetlag reported late meal timing for breakfast, early afternoon snack and dinner. Individuals with social jetlag also reported a higher intake of total calories (kcal), protein, total fat, saturated fat, cholesterol, and servings of meat and eggs and sweets in relation to those without social jetlag. Regarding the consumption during each meal of the day, participants with social jetlag had consumed more calories, saturated fat and cholesterol during dinner; more protein, total fat, saturated fat, and cholesterol during lunch; and more total fat and saturated fat during morning snack. In addition, individuals with social jetlag had a higher risk of inadequate consumption of total fat, saturated fat and cholesterol intake when compared with those without social jetlag. We conclude that social jetlag is associated with a poor diet and later meal times, which should be avoided in individuals with obesity-related chronic diseases. More studies are needed to confirm these findings.

## Introduction

The circadian clock system is an endogenous timing system that exists in a multitude of organisms to synchronize physiology and behavior with 24-h environmental cycles and to optimize energy balance, and thus survival [1]. The circadian clock programs daily rhythms and coordinates multiple behavioral and physiological processes, including activity, sleep, and eating [2], following reproducible oscillations across the 24-h day [3]. These rhythmic processes are governed by an internal circadian timing system but are also modulated by exogenous factors such as the light-dark cycle, social demands and work schedules [3,4].

The need to balance biological preferences with the requirements to meet pre-established working or study schedules can promote disruption of the circadian timing that alters sleep onset and wake up time between weekdays and weekends, producing a condition termed social jetlag [4,5]. Specifically, social jetlag reflects the difference in sleeping and waking hours on free days (determined by individual preference) and work days (established by social demands, that is, by schedules for working or studying) [4, 6]. For example, when a person sleeps from 11:00 p.m.to 05:00 a.m. on weekdays or workdays (mid-sleep time = 02:00 a.m.), and from 01:00 a.m. to 11:00 a.m. on weekends or free days (mid-sleep time = 06:00 a.m.), this results in a 4-h social jetlag (6:00–2:00). Evidence show that social jetlag is associated with an increased risk of development of obesity [6–9] and metabolic diseases [9–11]. We recently demonstrated that individuals with social jetlag (> 1 h) present a higher odds ratio of metabolically unhealthy obesity—condition in which the individual is obese (body mass index ≥ 30 kg/m^2^) and also presents high-risk values on three or more biomarkers for metabolic syndrome [8].

Despite the scarcity of studies explaining the possible role of social jetlag in the development of overweight and related diseases, studies have suggested that the circadian misalignment measured by social jetlag can compromise the maintenance of a healthy lifestyle, especially the behaviors related to tobacco consumption [10], physical activity [12] and feeding behavior [13, 14]. In this sense, studies have indicated that social jetlag could determine meal schedules, promoting food consumption at an inappropriate circadian time, as well as the intake of highly caloric or unhealthy food [5,14].

The lack of studies in this area makes evident the need to better explore the influence of social jetlag on feeding behavior, especially the composition of the diet and meal times in different populations, such as individuals with obesity and related diseases. Thus, this study investigated the association between social jetlag and food consumption at late meal timing in patients with obesity-related chronic diseases. We hypothesized that social jetlag is associated with eating meals at later times and eating more calories and fat, as well as an increased amount of food with a high content of these, among individuals with obesity-related chronic diseases.

## Material and methods

### Participants

This cross-sectional study involved volunteers with obesity-related chronic diseases who were attending outpatient clinics of the public health service of Uberlândia city, Minas Gerais State, Brazil. To be eligible to participate in the study, individuals had to have a confirmed medical pre-diagnosis of obesity, type 2 diabetes mellitus (TD2), systemic arterial hypertension or dyslipidemia (hypercholesterolemia, hypertriglyceridemia, or reduced HDL-C). The study was approved by the Ethics Committee of the Federal University of Uberlândia (protocol no. 005464/2015) and all volunteers provided a written informed consent form to participate in the study. Initially, the volunteers answered a questionnaire that assessed demographic aspects and health behaviors related to: sleep pattern, physical activity, alcohol intake, tobacco consumption and use of medicines. The methods used in this study have previously been described in detail elsewhere [8].

### Sleep pattern and social jetlag

For characterization of sleep pattern, participants were asked to report their usual bedtime, wake-up time, sleep-onset latency and usual sleep duration on weekdays and weekends for the past two weeks. Social jetlag was calculated following the protocol established by Wittman et al. [4], being the absolute difference between mid-sleep time on weekends and weekdays.

### Physical activity

The volunteers were asked about physical activity practice, type of exercise, weekly frequency, duration (in minutes of each section) and intensity of exercise. We calculated the total of minutes of physical activity per week by multiplying the frequency per week by the number of minutes per time. Participants were considered physically active if they performed at least 150 minutes of physical exercise per week [15].

### Anthropometric measures

Anthropometric measures were obtained by an expert team. Weight was determined by a scale to an accuracy of 0.1Kg (Welmy, São Paulo, Brazil) and height was measured using a stadiometer fixed to the wall with an accuracy of 0.1cm (Toledo Scale Corp., Toledo, Ohio). Height and weight were used to calculate the body mass index (BMI, kg/m^2^); a value above 25 kg/m^2^ or 30 kg/m^2^ was considered as overweight or obesity, respectively [16]. The neck circumference was measured by placing a tape horizontally around the neck (Plano Frankfurt), with the individual standing erect. A neck circumference ≥39 cm for men and ≥35 cm for women was considered high based on previous research [17]. The waist circumference (WC) was measured according to the standard proposed by the World Health Organization (WHO) [18] and values ≥ 102 cm for men or ≥ 88 cm for women were considered indicators of abdominal obesity.

### Dietary intake

The volunteers reported their food intake with the help of a team of three highly trained and experienced nutritionists in studies investigating food consumption. Dietary intake was assessed by a single 24-hour food recall (24h-FR) using the multiple pass method developed by the US Department of Agriculture (USDA) [19]. In this method dietary data was collected in five successive steps which makes it possible to increase the accuracy in obtaining food consumption information [19]. During this interview, the volunteers described with as much detail as possible the mealtimes and foods consumed on the previous day, providing quantitative and qualitative information such as brand names, food preparation methods and recipes for home-cooked foods. The day of the week of the 24h-FR evaluation was recorded by the researchers.

For daily intake we calculated: eating duration, defined as the time interval which reflects the length between the first and last caloric event each day [20]; caloric midpoint, which reflects the average time by which 50% of daily calories (kcal) had been consumed [13]; and the number of eating episodes per day. Events with ≥5 calories were considered an eating episode [20]. In order to verify consumption during the later hours of the day, we determined the total and the proportion (%) of calories consumed after 9:00 p.m. [8].

For the definition of each meal (breakfast, lunch, snacks, dinner or supper), we asked the participants about their perceptions and we also analyzed the type of food often present in the Brazilian diet. The pattern of a typical Brazilian diet includes three main meals: breakfast, lunch and dinner. Intermediate snacks are also frequent and tend to be eaten in the middle of the morning or afternoon [21]. To contemplate all possible patterns of intake we classified meal times as follows: breakfast, morning snack, lunch, early afternoon snack, late afternoon snack, dinner or supper.

The foods reported by the participants were converted into servings according to the energy content and were categorized into eight food groups: cereals, bread and pasta (150 calories); vegetables (15 calories); fruit (70 calories); beans (55 calories); meat and eggs (190 calories); milk and dairy products (120 calories); oils and fats (73 calories); and sweets (110 calories); following the established by the Adapted Food Pyramid [22]. The frequencies of daily intakes were considered to be “adequate” according to the recommendations of the Brazilian Food Pyramid [22] and for macronutrients, fiber and cholesterol intake, we used the recommended ranges established by the Institute of Medicine [23]. The energy and nutrient intake was analyzed using the Virtual Nutri Plus software and the same nutritionists team entered the data.

### Statistical analysis

All statistical analyses were performed with SPSS version 20.0 (SPSS Inc., Chicago, IL, USA), and p<0.05 was considered to be statistically significant. Initially, the volunteers were divided into two groups: patients with social jetlag (>1h) and without social jetlag (≤1h) [6]; this division was used to calculate descriptive statistics, a generalized linear model (GzLM) and logistic regression analyses. Normality of the data was tested using the Kolmogorov–Smirnov test. Categorical variables were summarized using frequencies and percentages, and continuous variables were summarized using means and standard deviations or median and interquartile intervals. Pearson’s chi-square test was used to compare proportion variables, and Student’s t-test or Mann–Whitney test for independent samples were used to compare continuous variables. GzLM was used to analyze the effect of social jetlag (independent variable) on meal times, energy, nutrient intake (carbohydrate, protein, total fat, saturated fat, cholesterol and fiber) and servings of each food group (dependent variables). Individual tests were done for each dependent variable using a Gamma or Linear distribution. Pairwise comparisons were performed using the Sidak sequential test.

In order to determine confounding factors, Pearson or Spearman’s correlation was carried out between the dependent variables and possible confounding variables (age, sex, family income, employment status, minutes of physical activity per week, BMI, mean sleeping duration, time of diagnosis of the chronic disease and use of insulin, antidepressants and/or sleeping pills). The variables correlated with each dependent variable in the Pearson or Spearman’s correlation (r> 0.20) were subjected to GzLM or multivariate logistic regression.

Multivariate logistic regression was used to predict the risk of ingestion above the recommendations of macronutrients, cholesterol, fiber and each food group by comparing the group with social jetlag (>1h) with those without social jetlag (≤1h). These results were expressed as the odds ratio with 95% confidence interval (CI).

## Results

This study included 792 individuals (73% female; age 55.9 ± 12.4 years) (Table 1). The group with social jetlag was younger (p <0.001), had a lower frequency of individuals with less than 12 years of schooling (p = 0.005) and a lower frequency of retirees (p <0.001). This group also reported higher consumption of alcohol units per week (p = 0.006); had a lower frequency of subjects who reported physical activity (p = 0.02); and had a higher prevalence of overweight participants (BMI > 25 kg/m^2^). Regarding the circadian data, the group with social jetlag reported going to sleep earlier on weekdays (p = 0.005) and later on weekends (p <0.001), waking up later at weekends (p <0.001), a longer duration of sleep on weekends (p <0.001) and a higher mean weekly sleep duration (p = 0.01) than those without social jetlag (Table 1).

**Table 1:** Demographics, employment status, health behaviors, anthropometric variables and sleep pattern according to absence of social jetlag (≤1h) or presence (>1h) (n = 792).

	All (n = 792)	Social jetlag ≤ 1h (n = 598)	Social jetlag > 1h (n = 194)	p*
Age (years)	55.9 ± 12.4	57.9 ± 11.6	49.8 ± 12.7	**<0.001**
Female (%)	581 (73.0)	433 (72.5)	148 (76.2)	0.30
Marital status–Married (%)	401 (51.0)	304 (51.0)	97 (50.0)	0.27
Family income–(U$ 553.0)	504 (63.0)	378 (63.2)	126 (65.0)	0.36
Education–< = 12 years	514 (69.0)	401 (67.0)	113 (58.2)	**0.005**
***Employment status***				
Day workers (%)	281 (35.9)	192 (32.5)	89 (46.3)	**<0.001**
Retired (%)	308 (39.3)	261 (44.2)	47 (24.4)	
Hours per week	40.8 ± 8.9	40.7± 9.3	41.0±8.1	0.80
***Health behaviours***				
Smoking status–Yes (%)	96 (12.0)	66 (11.0)	30 (15.4)	0.10
Alcohol intake–Yes (%)	220 (28.0)	155 (26.0)	65 (33.5)	0.10
Alcohol–Servings/week	2.0 [0.75–6.0]	2.0 [0.5–5.25]	4.0 [1.4–8.0]	**0.006**
Physical activity (PA)–Yes (%)	294 (37.0)	235 (39.3)	59 (30.4)	**0.02**
Minutes of PA/week^**Χ**^	180 [120–300]	180 [120–300]	180 [100–300]	0.34
***Anthropometric***				
BMI (kg/m^2^)	30.0 ± 6.3	29.4±6.4	30.1±5.6	0.13
Overweight (%)	575 (72.6)	421 (70.4)	154 (79.4)	**0.01**
Obese (%)	423 (53.4)	319 (53.3)	104 (53.6)	0.94
Abdominal obesity (%)^Ω^	564 (71.0)	423 (70.6)	141 (72.6)	0.58
High neck circumference (%)^Φ^	518 (65.0)	395 (66.2)	123 (63.4)	0.46
***Sleep pattern***				
Bedtime weekday (h:min)^↑^	22:12 [21:12–23:12]	22:25 [21:50–23:25]	22:00 [20:55–22:55]	**0.005**
Bedtime weekend (h:min) ^↑^	22:30 [21:30–24:00]	22:30 [21:30–23:30]	23:40 [22:30–24:50]	**<0.001**
Waketime weekday (h:min) ^↑^	06:00 [05:30–07:00]	06:00 [06:00–07:00]	06:00 [05:30–07:00]	0.339
Waketime weekend (h:min ^↑^	07:00 [06:00–08:30]	06:50 [06:00–08:00]	09:00 [08:00–10:00]	**<0.001**
Sleep duration weekday (h:min)	07:30 [06:30–09:00]	07:00 [06:00–08:25]	07:00 [06:00–08:25]	0.998
Sleep duration weekend (h:min)	08:00 [07:00–09:00]	07:42 [06:00–08:40]	08:25 [06:55–09:30]	**<0.001**
Mean sleeping duration (h)	07:30 ± 01:30	07.20 ± 01:40	07:30. ± 02:00	**0.01**

Note: Values are presented as mean and standard deviation for normally distributed data or as median (interquartile range) for non-normally distributed data.

*Pearson’s chi-square test was used to compare proportion variables and Student’s t-tests or Mann–Whitney test for independent samples were used in the comparisons for continuous variables. Bold value is statistically significant at *p* < 0.05.

^↑^Time is presented in 24-h clock time.is presented in 24-h clock time.

^Ω^Waist circumference≥102cm for men and≥88cm for women were considered abdominal obesity.

^Φ^Neck circumference ≥39cm for men and ≥35cm for women were considered high.

^Χ^Only those reported to perform physical activities.

We found a good representation of the days of the week and the weekend of 24-hour food recall collected in the present study: Monday = 21.0%, Tuesday = 27.7%, Wednesday = 23.5%, Thursday = 7.0%, Friday = 3.0% and Sunday = 17.8%.In this sense, we identified that 82.2% were evaluated on weekdays and 17.8% on weekends. No statistical difference was identified in the proportion of 24-hour recalls for weekdays and weekends among individuals with or without social jetlag(p = 0.87). Table 2 shows the estimated averages for food intake according to the absence (< = 1h) or presence (>1h) of social jetlag. After adjustments for age, sex, BMI, minutes of physical activity per week, mean sleeping duration and time since diagnosis of TD2, individuals with social jetlag consumed more total calories (p<0.001), protein (p<0.001), total fat (p = 0.002), saturated fat (p = 0.01), cholesterol (p<0.001), and servings per day of meat and eggs (p = 0.002) and sweets (p = 0.04), than those without social jetlag. The meal timing results revealed that participants with social jetlag ate their breakfast (p = 0.02), early afternoon snack (p<0.001) and dinner (p = 0.01) at later times than those without social jetlag (Table 2). In terms of daily intake, individuals with social jetlag also reported a longer eating duration (p = 0.03), higher calorie consumption after 9 p.m. (p<0.001), and a higher proportion of calories consumed after 9 p.m. (p<0.001) in relation to those without social jetlag (Table 2).

**Table 2:** Association of social jetlag on food consumption, meal timing and circadian intake according to absence of social jetlag (≤1h) or presence (>1h) (n = 792).

All (n = 792)	Social Jetlag ≤ 1h (n = 598)	Social Jetlag > 1h (n = 194)	
	Means ± SE	Means ± SE	p*
***Total Calories and nutrients***			
Calories (kcal/day)	1508.3 ± 20.2	1621.6 ± 38.1	**<0.001**
Carbohydrate (g/day)	182.0 ± 2.7	186.6 ± 5.0	0.42
Protein (g/day)	76.5 ± 1.4	85.1 ± 2.7	**<0.001**
Total fat (g/day)	52.6 ± 1.0	59.9 ± 2.1	**0.002**
Saturated Fat (g/day)	17.3 ± 0.4	19.4 ± 0.8	**0.01**
Cholesterol (mg/day)	246.2 ± 6.3	315.9 ± 14.1	**<0.001**
Fiber (g/day)	13.6 ± 0.2	13.3 ± 0.4	0.50
***Food groups (servings/day)***			
Cereals, breads and pasta	3.8 ± 0.07	3.9 ± 0.13	0.47
Vegetables	1.9 ± 0.06	2.0 ± 0.12	0.94
Fruits	3.2 ± 0.13	3.17 ± 0.24	0.95
Beans	1.7 ± 0.04	1.8 ± 0.09	0.38
Meat and eggs	2.2 ± 0.05	2.5± 0.10	**<0.002**
Dairy	1.6 ± 0.05	1.6 ± 0.09	0.68
Oils and fat	2.2 ± 0.08	2.2 ± 2.14	0.89
Sweets	1.4 ± 0.06	1.7 ± 0.12	**0.04**
***Meal timing***^***¥***^			
Breakfast (h:min)	7:20 ± 0:02	7:44 ± 0:04	**0.01**
Morning snack (h:min)	9:47 ± 0:04	9:45 ± 0:06	0.84
Lunch (h:min)	12:06 ± 0:18	12:12 ± 0:03	0.37
Early afternoon snack (h:min)	15:36 ± 0:02	15:54 ± 0:05	**<0.001**
Late afternoon snack (h:min)	17:22 ± 0:11	17:04 ± 0:18	0.45
Dinner (h:min)	19:42 ± 0:06	20:12 ± 0:11	**<0.001**
Supper (h:min)	20:31: 0:25	20:51 ± 0:05	0.76
***Daily intake***			
Eating duration∞ (h)	12:45 ± 0:06	13:09 ± 0:10	**0.03**
Caloric midpoint^∑^ (h:min)	13:41 ± 0:05	14:02 ± 0:10	0.08
Calories after 9 p.m.^**μ**^ (kcal/day)	105,1 ± 10.2	188,4 ± 26.9	**<0.001**
Calories after 9 p.m. ^**μ**^ (% of total EI)	6.2 ± 0.5	10.5 ± 0.9	**<0.001**
Number of eating episodes	4.2± 0.4	4.1± 0.4	0.21

*GzLM analyses were adjusted for age, sex, BMI, minutes of physical activity per week and mean sleeping duration. Bold value is statistically significant at *p* < 0.05.

^¥^Total individuals who reported performing each of the meals or snacks: breakfast (n = 747); morning snack (n = 237); lunch (n = 779); early afternoon snack (n = 604); late afternoon snack (n = 64); dinner (n = 733); and supper (n = 128).

∞Length between the first and last caloric event each day.

^∑^Caloric midpoint: the average time at which 50% of daily calories had been consumed.

^**μ**^Caloric intake reported after 9 p.m. was related to the food consumption of the dinner and / or supper, depending on the consumption schedules of the individuals.

EI: energy intake.

**Fig 1:**
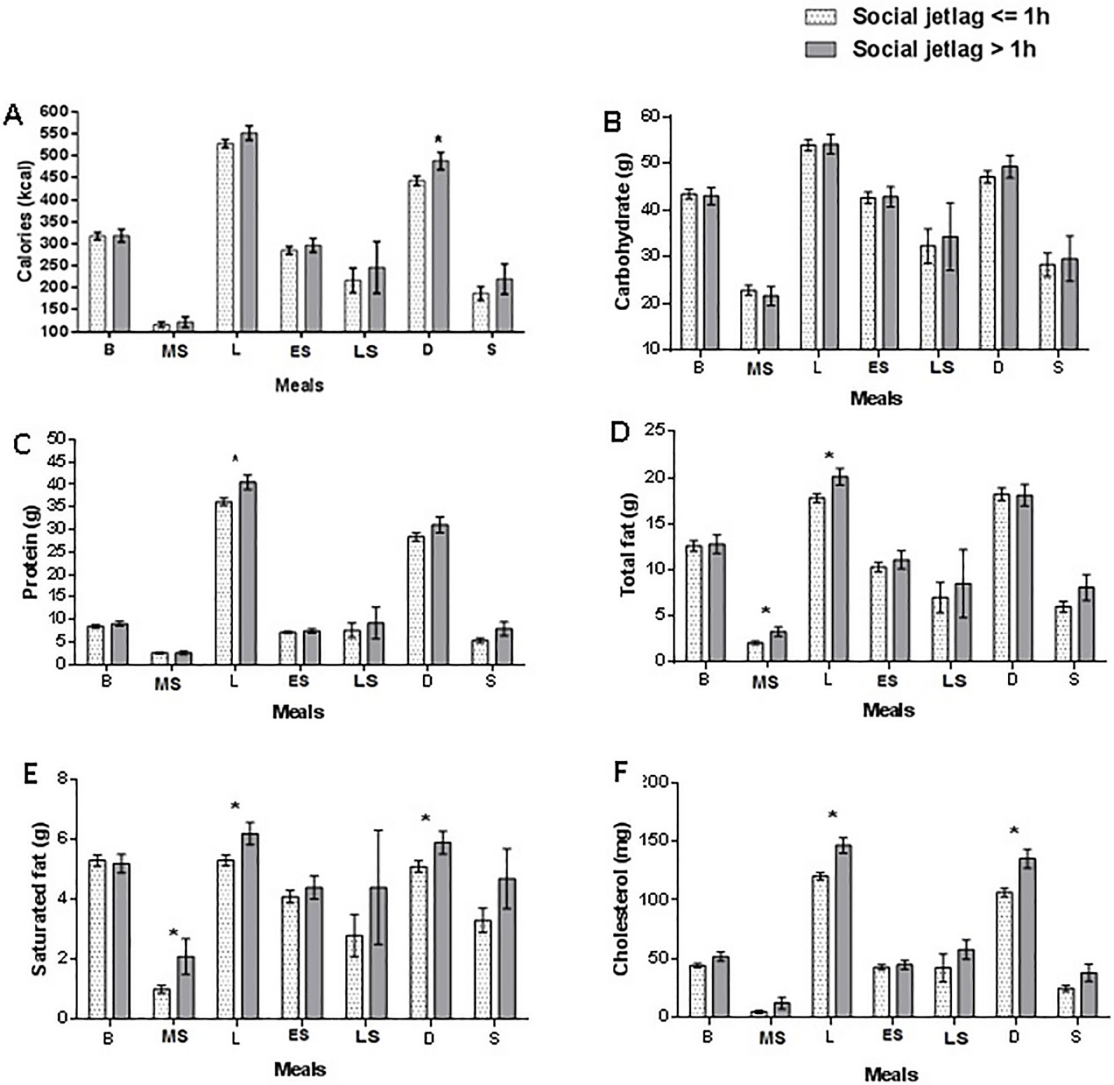
Association of social jetlag on distribution of calories and macronutrients at each meal or snack along of the day. Note: A. Calories (kcal): group with social jetlag had a higher calorie (kcal) intake at dinner (443.4 ± 10.5 vs. 488.4 ± 19.8; p = 0.03). B. Carbohydrate (g): no association of social jetlag. C. Protein (g): group with social jetlag had a higher protein (g) intake at lunch (36.2 ± 0.80 vs 40.5 ± 1.60; p = 0.01). D. Total fat (g): group with social jetlag had a higher total fat intake (g) in the morning snack (p = 0.02) and lunch (p = 0.03). E. Saturated fat (g): group with social jetlag had a higher saturated (g) fat intake in the morning snack (1,0g ± 0.13 vs. 2.1g ± 0.59; p = 0.02); lunch (5.3g ± 0.18 vs. 6.2g ± 0.37;p = 0.03) and dinner (5.1g ± 0.19 vs. 5.9g ± 0.38; p = 0.03). F. Cholesterol (mg) group with social jetlag had a higher cholesterol intake (mg) at lunch (120.4 ± 3.1 vs. 146.5 ± 6.6 p<0.001) and dinner (106.5 ± 3.7 vs. 135.3 ± 8.0; p<0.001). *GzLM analyses were adjusted for age, sex, BMI, minutes of physical activity per week and mean sleeping duration. Values are presented as mean and standard error (SE).Total individuals who reported performing each of the meals or snacks: breakfast (n = 747); morning snack (n = 237); lunch (n = 779); early afternoon snack (n = 604); late afternoon snack (n = 64); dinner (n = 733); and supper (n = 128). Caloric intake reported after 9 p.m. was related to the food consumption of the dinner and / or supper, depending on the consumption schedules of the individuals. B: breakfast; MS: morning snack; L: lunch; ES: early afternoon snack; LS: late afternoon snack; D:dinner; S:super.

When we analyzed the distribution of calories and macronutrients between meals, the group with social jetlag had a higher calorie (kcal) intake at dinner (p = 0.03), protein intake (g)at lunch (p = 0.01), total fat (g) intake in the morning snack (p = 0.02) and lunch (p = 0.03),higher saturated fat (g) intake in the morning snack (p = 0.02), lunch (p = 0.03) and dinner (p = 0.03);and higher cholesterol (mg) intake at lunch (p<0.001) and dinner (p<0.001) -when compared to those without social jetlag (Fig 1).

The results of the adjusted logistic regression indicated a higher risk of consumption above recommendations for total fat (odds ratio [OR] = 1.3, CI = 1.1–1.9; p = 0.03], saturated fat (OR = 1.2, CI = 1.1–2.0; p = 0.01) and cholesterol intake (OR = 1.8, CI = 1.3–2.6; p<0.001) in individuals with social jetlag when compared to those without social jetlag (Table 3).

**Table 3:** Odds ratio (OR) for inadequate food consumption according to presence (>1h) or absence of social jetlag (≤1h –reference group) (n = 792).

Food intake	Inadequate food consumption^∑^	OR (IC 95%)*	p
**Food groups (servings/day)**			
Cereals, bread and pasta	<5 or >9 servings/day	1.0 (0.5–1.1)	0.23
Vegetables	<4 servings/day	1.2 (0.6–2.3)	0.60
Fruits	<3 servings/day	1.0 (0.7–1.6)	0.80
Beans	<1 servings/day	1.2 (0.8–1.7)	0.23
Meat and eggs	<1 or >2 servings/day	1.0 (0.7–1.4)	0.81
Milk and dairy	<3 servings/day	0.7 (0.4–1.4)	0.35
Oils and fats	>2 servings/day	1.2 (0.8–1.7)	0.22
Sweets	>1 servings/day	1.0 (0.6–1.5)	0.94
**Nutrients**			
Carbohydrate (%EI)	<45 or >65% (EI)	1.1 (0.8–1.6)	0.46
Protein (%EI)	<10 or >35% (EI)	1.4 (0.5–4.2)	0.46
Total fat (%EI)	>30% (EI)	1.3 (1.1–1.9)	**0.03**
Saturated fat (%EI)	>10% (EI)	1.2 (1.1–2.0)	**0.02**
Cholesterol (mg/day)	>300 mg/day	1.8 (1.3–2.6)	**<0.001**
Fiber (g/day)	<14g/1000 calories/day	1.7 (0.9–3.3)	0.09

^**∑**^From Philippi ST, 1999; IOM, 2005.

*Multivariate logistic regressions analysis adjusted model for age, sex, BMI, minutes of physical activity per week and mean of sleep duration. Bold value is statistically significant at *p* < 0.05. EI: energy intake.

## Discussion

This study evaluated the association between social jetlag and food consumption at late meal timing in patients with obesity-related chronic diseases. The participants with social jetlag (>1h) had a higher intake of calories, protein, total fat, saturated fat, cholesterol, and servings of meat, eggs, and sweets in relation to those without social jetlag (< = 1h). They also reported a longer eating duration and later meal timing for breakfast, early afternoon snack and dinner. Regarding the composition of each meal, individuals with social jetlag ate more calories, saturated fat and cholesterol during dinner; protein, total fat, saturated fat, and cholesterol during lunch; and total fat and saturated fat during the morning snack. In addition, individuals with social jetlag had a higher risk of inadequate consumption of total fat, saturated fat and cholesterol intake when compared with those without social jetlag (≤1h). These results suggest that social jetlag is associated with a poor diet and later meal times, which should be avoided in individuals with obesity-related chronic diseases. More studies are needed to confirm these results and to describe the possible mechanisms that explain these associations.

Until now, few studies have evaluated the effects of social jetlag on food intake. A study on rats showed that the combination of social jetlag and a cafeteria diet (rich in fat and carbohydrates) led the animals to overeat and also increased their bodyweight and the number of criteria indicating metabolic syndrome [5]. In humans, a study conducted by our research group on Brazilian undergraduate students found that social jetlag was negatively associated with the consumption of beans [14], a typical constituent of the Brazilian diet that has been associated with protection against overweight [24]. Almoosawi et al., [25] studied a nationally representative sample of apparently healthy adults from the United Kingdom and found a negative association between the degree of social jetlag and the healthy dietary pattern scores. In the present study, individuals with social jetlag reported a higher intake of meat and eggs, sweets, calories, protein, total fat, saturated fat and cholesterol (Table 2), and had a risk of consumption above the recommendations for total fat, saturated fat and cholesterol when compared to those without social jetlag(Table 3). It is widely recognized that the control of these food groups and nutrients is critical for biomarker levels related to obesity-related chronic diseases, such as glucose, triglycerides and HDL-cholesterol [26–28]. Thus, it will be important to confirm whether social jetlag can in some way influence food consumption and the mechanisms involved. Such findings may make it imperative that the dietary treatment of individuals with obesity-related chronic diseases also considers chronobiological aspects of circadian sleep rhythms and food consumption.

The widespread availability of electrical lighting and the current habit of using media such as the television, computer or mobile phone especially prior to bedtime [1,29], which can extend wakefulness activities far into the night, thus altering the timing of wakefulness[1] and increasing the “window to eat”, or more specifically the eating duration (the length between the first and last caloric event each day)[20]. Recently, focusing on eating duration has emerged as a potential strategy for avoiding major dietary changes by imposing a time-restricted feeding regimen in which all caloric intake occurs within an interval <12-h every day [20,30]. This approach appears to impart both preventative and therapeutic effects on metabolic diseases in experimental animals [31,32] and body weight in humans [20]. An unexpected and interesting result found in the present study was that participants with social jetlag reported a longer eating duration (Table 2), which may suggest a greater availability of time to eat. However, McHill et al. [13] evaluated college-age individuals (age range 18–22 years) and found no association between social jetlag and eating duration; however, these authors found a positive association between eating duration and the percentage of calories consumed 4h before dim-light melatonin onset (DLMO), a physiological marker of the “biological night”. It has been suggested that eating closer to or after DLMO may be decreased thermic effect of food, which would contribute to a positive energy balance and weight gain [13]. Nevertheless, it should be noted that eating duration does not consider the time of each meal. For example, individuals who do not eat breakfast or who initiate their food routine at a very late time could have an eating duration of less than 12 hours, but they could drag eating times away from synchronization with the environmental light cycle. Thus, the ideal eating duration should be in line with daylight hours, such that individuals eat with favorable digestive and metabolic activity [20,33]. Further studies–including in humans–are needed to better understand the influence of time-restricted feeding on obesity and related diseases, contemplating both eating duration and meal times, as well as the composition and distribution of macronutrients among meals.

In the present study, participants with social jetlag ate their breakfast, early afternoon snack and dinner at a later time than those without social jetlag (Table 2). It has been suggested that later food intake does not appear to be particularly satiating and can result in greater overall daily intake[34,35]. There is also evidence of studies in laboratory conditions [36–38] and shift workers [39] that indicate a decrease in satiety-related hormones in situations of circadian misalignment. These mechanisms help us to understand why individuals with social jetlag also reported higher consumption of total calories, proteins, total fat, saturated fat and cholesterol,and in particular, higher calorie consumption after 9 p.m., than those without social jetlag (Table 2). Taken together, this evidences suggests that social jetlag may be a mediator that leads individuals to eat later, which may decrease satiety and increase appetite, favoring weight gain and compromising the control of important biomarkers for the control of obesity-related chronic diseases.

Eating at a later time could alsoinfluence the success of weight-loss therapy [20,40] and metabolic control [41,42]. Garaulet et al. [40] showed that late lunch eaters (>15h) lost less weight and displayed a slower weight-loss rate during the 20 weeks of treatment than early lunch eaters. Interestingly, the timing of the early afternoon snack in the present study was around 15h; however, the group with social jetlag reported eating this snack later (Table 2). A recent study also found that meal intake at night comprised of low glycemic ingredients–and, coincidentally, eaten at around the same time that individuals with social jetlag ate dinner in the present study (Table 2)–contributes to higher glucose excursions and concomitantly higher insulin levels throughout the night, compared with an equivalent meal in the morning [41]. These results could explain the ability of the timing and composition of food to alter peripheral tissue clocks, leading to internal disruption [1] with misalignment between gene transcription cycles within metabolic tissues and the behavioral cycle (of fasting and feeding) [43], which could alter energy expenditure [38] and have adverse metabolic effects [38,44].

The data on the composition of each meal revealed that individuals with social jetlag consumed calories, saturated fat and cholesterol at dinner; more protein, total fat, saturated fat, and cholesterol at lunch; and more total fat and saturated fat during their morning snack (Fig 1). Individuals with social jetlag also hada higher risk of consumption beyond the recommendations for total fat, saturated fat and cholesterol intake than those without social jetlag (< = 1h) (Table 3). In animals, a high-fat diet leads to changes in the expression and cycling of canonical circadian clock genes (nuclear receptors that regulate clock transcription factors) and clock-controlled genes (involved in fuel utilization in the hypothalamus, liver and adipose tissue) [2]. A high fat diet also led to increased diurnal activity (rest phase for rats) and decreased nocturnal activity (activity phase for rats) accompanied by obese-like body weights and high plasma triglyceride levels [45]. Moreover, a low-carbohydrate, high-protein diet can alter the circadian expression of molecular clock genes and affects glucose homeostasis in animals [46]. Interestingly, Esptia-Bautista et al. [5] showed that access to a cafeteria diet only during the rest period did not lead to weight or metabolic changes related to the diagnosis of metabolic syndrome. Only associated circadian misalignment in the social jetlag model and cafeteria diet consumption promotedthe criteria for establishment of the metabolic syndrome. The sum of these results may favor the notion that there is a “double track” within this theme, such that the composition of the diet changes the physiological rhythms, especially of the peripheral tissues; and the circadian misalignment caused by social jetlag alters food choices and the composition and distribution of food throughout the day.

This study has some limitations. First, its cross-sectional design does not allow the establishment of causal relationships, although it could be considered sufficient to address the main objective of the study. Some evaluations were carried out using questionnaires, which, although validated in other studies, are subjective in nature and depend on an individual’s memory and motivation. For this reason we used an expert team of researchers with experience in studies with similar methodology. Also, the analysis of the food intake only by one day of food recall may possibly compromise the accuracy of data on calories, macronutrients [47] and micronutrients [48]. Therefore, we had a good representation of weekdays and weekends between the food recalls of individuals evaluated, which has been shown to improve accuracy of the data [49]. We also found a relatively low total calorie intake, which may be due to the bias of underreporting presented by the subjective methods of food consumption assessment [47]. To avoid this, we used strategies that could increase volunteer engagement and prevent omission or forgetfulness of information [19]. Finally, we did not evaluate the pattern of physical activity with methods that assess the time when the individual exercises, and this issue could supposedly impact meals. Future studies should question the timing of physical activity practices to better understand the relationship between social jetlag and meal times.

## Conclusion

In summary, we conclude that social jetlag is associated with the schedules of meals and snacks, dragging consumption to later periods of the day. In addition, the presence of social jetlag is related to the total consumption and distribution of calories and macronutrients, especially proteins, total fats, saturated fat and cholesterol, throughout the day. This evidence compliments that found in experimental studies and laboratory conditions, which has revealed a negative impact of circadian misalignment on food behavior. It is necessary to carry out new studies to verify whether the synchronization of feeding behavior, as well as of sleeping and waking hours, with environmental cycles could minimize the impacts of these factors on health, especially in individuals in which obesity-related chronic diseases are already present.

## Supporting information

S1 File The database file for this manuscript. (XLS)
